# Contrasting income-based inequalities in incidence and mortality of breast cancer in Korea, 2006-2015

**DOI:** 10.4178/epih.e2024074

**Published:** 2024-09-11

**Authors:** Jinwook Bahk, Hee-Yeon Kang, Young-Ho Khang, Kyunghee Jung-Choi

**Affiliations:** 1 Department of Public Health, Keimyung University, Daegu, Korea; 2 Department of Cancer Control and Population Health, National Cancer Center Graduate School of Cancer Science and Policy, Goyang, Korea; 3 Department of Health Policy and Management, Seoul National University College of Medicine, Seoul, Korea; 4 Institute of Health Policy and Management, Seoul National University Medical Research Center, Seoul, Korea; 5 Department of Environmental Medicine, Ewha Womans University College of Medicine, Seoul, Korea

**Keywords:** Breast cancer, Health inequities, Incidence, Mortality, Korea

## Abstract

**OBJECTIVES:**

Breast cancer incidence and mortality rates in Korea are increasing. This study analyzed income-based inequalities in the incidence and mortality of women breast cancer from 2006 to 2015, using national data that covered all Korean women.

**METHODS:**

We used the National Health Information Database from 2006 to 2015. For women aged 20 and older, the age-standardized incidence and mortality rates of breast cancer per 100,000 by income quintile per year were calculated using the direct method. The rate ratio and rate difference (RD) of the age-standardized incidence and mortality rates of breast cancer per 100,000 between the top and bottom income quintiles were calculated as relative and absolute measures for inequalities.

**RESULTS:**

When comparing 2006 and 2015, both the incidence and mortality rates of breast cancer increased. The lowest income quintile experienced higher mortality rates despite having lower incidence rates. In 2015, the income-based RD in incidence and mortality rates between the highest and lowest income quintiles (Q1-Q5) was -19.9 (95% confidence interval [CI], -24.3 to -15.5) and 4.4 (95% CI, 2.9 to 5.8), respectively. Throughout this period, there was no statistically significant trend in income-based disparities in breast cancer incidence and mortality. The age-specific contributions to the absolute magnitude of inequality (RD) in incidence and mortality were more pronounced among middle-aged women than among older women.

**CONCLUSIONS:**

This study found that breast cancer in Korea exhibited pro-rich inequalities in mortality despite pro-poor inequalities in incidence. More equitable policies for screening and treatment of breast cancer are needed.

## GRAPHICAL ABSTRACT

[Fig f2-epih-46-e2024074]

## Key Message

This study found that breast cancer in Korea exhibited pro-rich inequalities in mortality despite pro-poor inequalities in incidence. More equitable policies for screening and treatment of breast cancer are needed.

## INTRODUCTION

Globally, breast cancer is the most frequently diagnosed cancer among women and the leading cause of cancer death [1]. Korea is no exception, with high incidence and mortality rates of breast cancer. Since 2005, breast cancer has been the second most common cancer diagnosis after thyroid cancer, with a consistently increasing trend [2]. Moreover, breast cancer mortality in Korea has been rising and has ranked among the top 6 causes of cancer death in women since the 1980s [2]. Despite these increasing trends in both incidence and mortality, the absolute levels of these indicators, particularly breast cancer mortalities, remain relatively low compared to other developed countries [3]. The low mortality rate of breast cancer in Korea can be attributed to relatively high screening rates [4], early diagnosis [5], and high survival rates [6].

Breast cancer, often referred to as a “cancer of affluence,” exhibits a pro-poor incidence in developed countries such as the United States and European nations, meaning it has a lower incidence in lower-income groups—a pattern indicating a positive association [7,8]. This contrasts with many other diseases, which typically show a pro-rich incidence, where lower incidence rates are observed in higher-income groups, reflecting a negative association. However, the patterns of breast cancer mortality inequalities vary by country. While pro-poor mortality patterns are frequently observed [9], some studies report either a negative association or no significant relationship between breast cancer mortality and socioeconomic indicators [10-13]. A previous study highlighted a shift in the direction of breast cancer mortality inequality trends in Korea, moving from pro-poor to pro-rich associations in the early 2000s [13-15]. This shift was potentially influenced by changes in the inequality of incidence from a positive to a negative direction [15]. Although several prior Korean studies have examined inequalities in breast cancer incidence and mortality [13-15], none have concurrently explored the trends of both incidence and mortality inequalities. In this study, we analyzed national data covering the entire population of Korean women to examine the trends of breast cancer incidence and mortality inequalities from 2006 to 2015, observing any changes in directional inequality.

## MATERIALS AND METHODS

### Data

Data were constructed using the National Health Information Database (NHID) from 2006 to 2015. This database is provided by the National Health Insurance Service (NHIS), which covers all Korean nationals living in Korea [16]. The study subjects were women aged 20 years or older. To accurately identify the incidence of breast cancer, we utilized the application records from the rare and intractable disease (RID) registration for breast cancer (C50). This registration enhances health insurance benefits for cancer patients, thereby reducing their out-of-pocket expenses [17]. The application record of the RID registration is recognized as the most reliable method for determining cancer incidence using the NHID in Korea [17]. An incident case of breast cancer was defined as any woman who, within the first year, was either hospitalized or had at least 2 outpatient visits with a primary or secondary diagnosis of breast cancer (C50). This was among those who benefited from the reduced co-payment policy for breast cancer each year from 2006 to 2015 and who had no prior history of reduced co-payment benefits for breast cancer in the preceding 5 years. However, since the reduced co-payment policy was initiated in 2005, we excluded any cases from previous years that had a treatment record for breast cancer.

Deaths from breast cancer were obtained by using the resident registration number to link the NHID to mortality registration data from Statistics Korea for the years between 2006 and 2015. Statistics Korea’s mortality registration data cover all deaths in Korea. NHIS premiums were used as a proxy for income. NHIS data were structured according to the insured and their dependents rather than being based on household members. Given this limitation, income was measured at the level of insured and their dependents. The equivalized income was calculated by dividing the NHIS premium paid by the insured by the square root of the number of insured individuals and dependents. Income quintiles were then determined annually for the entire NHID population, based on this equivalized household income [18].

Between 2006 and 2015, a total of 189,800,244 women aged 20 years or older were analyzed. During this study period, 121,071 new cases and 18,891 deaths were recorded. The annual data on population size, incidence, and breast cancer mortality across different income quintiles from 2006 to 2015 are detailed in Supplementary Material 1.

### Statistical analysis

The age-standardized incidence and mortality rates of breast cancer per 100,000, stratified by income quintile for each year, were calculated using the direct method with the total population across all years as the standard population. We also computed the mortality-to-incidence ratios (MIRs) using these age-standardized rates. We calculated the rate ratio (RR) and rate difference (RD) for the age-standardized incidence and mortality rates of breast cancer per 100,000 between the highest (Q5) and lowest (Q1) income quintiles, with the highest quintile serving as the reference group. These calculations provided relative and absolute measures of inequalities. The RDs and RRs for the incidence and mortality of breast cancer in other income quintiles (Q2, Q3, and Q4) compared to the highest quintile (Q5) from 2006 to 2015 are detailed in Supplementary Materials 2 and 3. Additionally, we presented the RR and RD for the incidence and mortality of breast cancer by age group (20-24, 25-29, …, ≥ 85 years) for the most recent year, 2015. The incidence and mortality rates of breast cancer by age group and income quintile for the years 2006, 2010, and 2015 are shown in Supplementary Materials 4-6. These analyses were conducted using the PROC STDRATE procedure in SAS software. A time trend analysis of the RR and RD from 2006 to 2015 was performed using weighted least squares regression to fit a linear model, incorporating standard errors as weights. All analyses were carried out using SAS version 9.4 (SAS Institute Inc., Cary, NC, USA).

### Ethics statement

This study was approved by the NHIS of Korea (No. NHIS-2019-1-103) and the Seoul National University Hospital Institutional Review Board (IRB No. E-2002-016-1098).

## RESULTS

**Figure 1.**
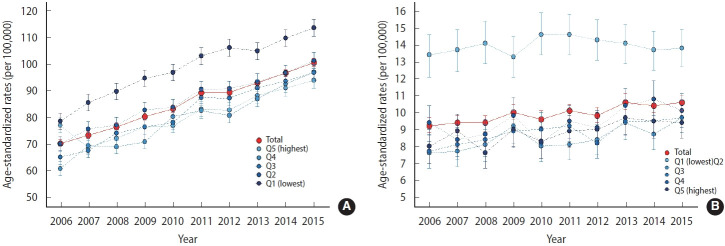
The age-standardized incidence (A) and mortality (B) rates of breast cancer by income quintile between 2006 and 2015 in Korea.

Figure 1 and Supplementary Material 7 present the age-standardized incidence and mortality rates of breast cancer per 100,000 population in Korea from 2006 to 2015. During this period, the overall age-standardized incidence rate of breast cancer increased. Specifically, in the lowest income group, the age-standardized incidence rose from 76.9 (95% confidence interval [CI], 74.0 to 79.9) in 2006 to 93.8 (95% CI, 90.9 to 96.8) in 2015, marking an increase of 16.9 per 100,000. This rising trend was consistent across all income quintiles, with increases of 16.9, 36.5, 32.0, 31.6, and 35.2 for the bottom to top income quintiles, respectively. The second income quintile group (Q2) saw the largest absolute increase in breast cancer incidence from 2006 to 2015, whereas the bottom income quintile experienced the smallest absolute increase. Over the decade, the highest incidence rates were observed in the top income quintile group. Since 2007, a consistent graded pattern has been noted among the Q1, Q4, and Q5 groups. However, the Q2 and Q3 groups showed slight deviations from this pattern between 2007 and 2013. Notably, in 2009, the incidence rate for the Q2 group was 70.7 (95% CI, 68.0 to 73.4), which was lower than that of Q1, which stood at 76.3 (95% CI, 73.5 to 79.1).

In contrast, Figure 1 illustrates that the trend in breast cancer mortality over the past decade has been less pronounced. Between 2006 and 2015, there was a slight increase in the absolute mortality level in the second income quintile (Q2), rising from 7.6 (95% CI, 6.7 to 8.6) per 100,000 in 2006 to 9.7 (95% CI, 8.8 to 10.6) in 2015. A similar slight increase was observed in the third income quintile (Q3), where mortality rates rose from 7.7 (95% CI, 6.7 to 8.7) per 100,000 in 2006 to 9.7 (95% CI, 8.8 to 10.7) in 2015. No significant differences were noted in other income quintiles. The incidence in the Q1 group has consistently been the highest since 2006. From Q2 to Q5, the mortality rates were consistently lower than those in Q1; however, a clear graded pattern was not observed. The MIRs, which combine incidence and mortality rates, indicated a decreasing trend in 2015 compared to 2006, with the Q1 group having the highest and the Q5 group having the lowest rates (Supplementary Material 7).

Table 1 presents the RD and RR for breast cancer incidence and mortality across the highest and lowest income quintiles. The RD for the incidence rate was -17.0 (95% CI, -21.1 to -12.9) in 2007 and -19.9 (95% CI, -24.3 to -15.5) in 2015. Regarding breast cancer mortality, the RD was 5.4 (95% CI, 3.8 to 7.0) in 2006, 6.4 (95% CI, 4.8 to 7.9) in 2010, and 4.4 (95% CI, 2.9 to 5.8) in 2015. The RR for breast cancer mortality was 1.87 (95% CI, 1.61 to 2.17) in 2008 and 1.44 (95% CI, 1.27 to 1.64) in 2014, indicating higher mortality rates in the bottom income quintile compared to the top quintile.

Table 2 contains the RDs and RRs for the relationships between income quintiles (Q1 vs. Q5) and breast cancer incidence and mortality by age group in Korea for the year 2015. The age group that contributed most significantly to the RD in breast cancer incidence and mortality between the highest and lowest income quintiles was 50-54 years old. For this group, the RD was -50.6 (95% CI, -68.1 to -33.0) for incidence and 13.5 (95% CI, 8.1 to 19.0) for mortality. Additionally, the RDs and RRs for incidence in the 35-39, 40-44, 45-49, and 65-69 age groups were statistically significant, indicating a higher breast cancer incidence in the highest income quintile. Regarding mortality, the 35-39 and 55-59 age groups also showed statistically significant RDs and RRs, suggesting lower mortality rates in the highest income quintile.

## DISCUSSION

This study demonstrated that both the incidence and mortality rates of breast cancer increased from 2006 to 2015. The incidence rates consistently showed a pro-poor inequality when comparing the highest and lowest quintiles (Q5 vs. Q1), whereas the mortality rates exhibited a pro-rich bias. Throughout this period, there was no statistically significant trend or shift in the direction of inequality in breast cancer incidence and mortality. The contribution to the inequality of both incidence and mortality was more pronounced among middle-aged women than among older adults.

A previous study indicated a shift in the trends of breast cancer mortality inequality in Korea, moving from pro-poor to pro-rich disparities in the early 2000s [15]. The authors explored factors that might have influenced this shift and raised the possibility of a similar trend in incidence inequality, although the studies referenced had relatively small sample sizes [19-21]. Utilizing national data that encompassed all women in Korea, this study found consistent pro-poor inequalities in breast cancer incidence from 2006 to 2015, diverging from the mortality pattern observed. Moreover, the trend of pro-poor inequality in breast cancer incidence remained stable throughout the period. This result is consistent with recent research using the Korea Central Cancer Registry (KCCR) database [22]. Additionally, this persistent unfavorable trend in breast cancer incidence among the higher socioeconomic position (SEP) group was remarkably consistent across multiple countries [7,8,23-29].

Reproductive factors such as early menarche, delayed age at first full-term pregnancy, less breastfeeding, and late menopause, along with behavioral factors such as a high-fat diet, obesity, smoking, and oral contraceptive usage, are well-known risk factors for breast cancer incidence. This suggests that the distribution of these factors could contribute to pro-poor inequalities [30,31]. For instance, in Korea [32], as in Western countries [33,34], the age at first birth was less favorable in the higher education group. However, Korean studies have also reported contradictory findings regarding the relationship between SEP and risk factors for breast cancer. For example, age at menarche or late menopause was not significantly correlated with SEP in Korea [35,36]. Previous studies have also indicated that breastfeeding duration was shorter, and the prevalence of obesity, oral contraceptive use, and smoking was higher in the lower SEP group [37-40]. This complex situation may contribute to the unclear graded pattern of breast cancer incidence. To better understand the pro-poor inequalities in breast cancer incidence, further research is needed, taking into account the incubation period between exposure to risk factors and the development of breast cancer.

Breast cancer screening plays a significant role in influencing the disparities in breast cancer incidence and mortality across different SEP groups. It not only potentially reduces mortality through early detection but also may increase detection rates, contributing to a higher incidence rate [41,42]. The National Cancer Screening Program for breast cancer, launched in 1999 in Korea, initially provided free screening solely to the medical aid beneficiary group (a subgroup of the lowest income quintile in this study). It was subsequently expanded to cover national health insurance beneficiaries in the lowest 50% of income by 2006 [43]. Despite the increase in the breast cancer screening rate from 40.6% in 2006 to 61.2% in 2015 [4], women in the lower SEP group were less likely to undergo breast cancer screening [44]. This suggests that women from lower socioeconomic backgrounds may face a higher risk of delayed diagnoses and presenting with advanced tumor stages [22].

Breast cancer in Korea exhibits the highest mortality and MIRs in the lowest income group, despite a pro-poor inequality in incidence. Additionally, a definitive social gradient in mortality is not observed, except in the lowest income group. If the occurrence of a disease naturally led to death, the magnitude and direction of inequality in mortality would correspond to those in incidence. However, during the progression from breast cancer onset to death, factors that disproportionately disadvantage the low-income class could intervene, leading to a reversal in the direction and a weak social gradient of mortality inequality. This reversal pattern was similar to that observed in the United States [11,45], but contrasts with the trend in European countries, where positive associations between SEP and both mortality and incidence have been found [8,9]. In European countries, both breast cancer incidence and mortality were higher in the higher SEP group, although the survival rate was also higher in this group. These contrasting patterns from Europe, Korea, and the United States could lead to the assumption that women with lower SEP in Korea and the United States are unable to overcome the comparative disadvantage of survival by SEP despite the comparative advantage of incidence. Survival rate was related to the stage at diagnosis, timely treatment, treatment completion, or quality of care [46]. A recent study using 2007-2017 data from the KCCR showed that the risk of distant-stage diagnosis in breast cancer was higher in the lower-income group [22]. The quality assessment result for breast cancer treatment practice in the metropolitan area was greater than that in the province [47]. Survival rates in blue-collar workers with breast cancer were lower than in white-collar occupations [48], which was consistent with the result of this study showing pro-rich inequalities in MIRs.

The findings of this study should be interpreted with caution for several reasons. First, the study utilized data from the NHID, rather than the KCCR. The KCCR data, which achieved a 98.2% completeness rate for cancer incidence in 2015 through various methods, including hospital-based and population-based registries, as well as medical chart reviews [49], is generally more comprehensive. However, the sensitivity of the breast cancer definition based on the NHID, when compared to KCCR data, was still relatively high at 98.1% [17]. Additionally, a study on income inequality and breast cancer incidence using KCCR data revealed a similar pattern to that found in this study [22]. Second, this study used insurance premiums as a proxy for income because the NHID does not provide actual income data. The appropriateness of using premiums as an income indicator has been debated, particularly due to the different levying systems for employees and the self-employed. A previous study investigated whether insurance premiums could effectively indicate socioeconomic status and its impact on health inequality despite these challenges [18]. It found that the disparity in life expectancy between the lowest and highest income quintiles was most pronounced when income groups were classified without distinguishing between different types of NHIS beneficiaries. This study adopted these classification methods for income groups. Furthermore, since insurance premiums are used as a proxy for income, any changes in the premium charging system, independent of actual income changes, could potentially affect the classification of income groups. However, there were no significant changes in the insurance premium charging system from 2006 to 2015, making it unlikely that such changes significantly influenced our findings. Third, the absence of follow-up data on the breast cancer patients in this study meant that we could not analyze survival rates or the broader causes of death beyond breast cancer itself. Fourth, various factors potentially related to the incidence of breast cancer, such as reproductive factors, hormone replacement therapy, and lifestyle factors, need to be considered to fully explain the observed trend of income inequality in breast cancer incidence. Similarly, further research is needed to identify the factors contributing to mortality inequalities in breast cancer, including stage at diagnosis, tissue characteristics, disease severity, time to treatment, and quality of care. Lastly, we used RR and RD instead of the slope index of inequality (SII) and the relative index of inequality (RII). While RR and RD provide a straightforward comparison between only 2 groups, SII and RII encompass all SEP groups and their sizes. Despite this, RR and RD offer advantages such as intuitive clarity, ease of computation, and reliance on actual observations from both groups. Previous research has also shown that the values of SII and RII are similar to those of RR and RD [50].

Nonetheless, our study utilized a single dataset, the NHID data, which enabled us to concurrently examine the patterns of breast cancer incidence and mortality. We discovered that in Korea, breast cancer mortality exhibited pro-rich inequality, while incidence showed pro-poor inequality. This underscores the need for more equitable policies in the screening and treatment of breast cancer.

## Figures and Tables

**Figure f2-epih-46-e2024074:**
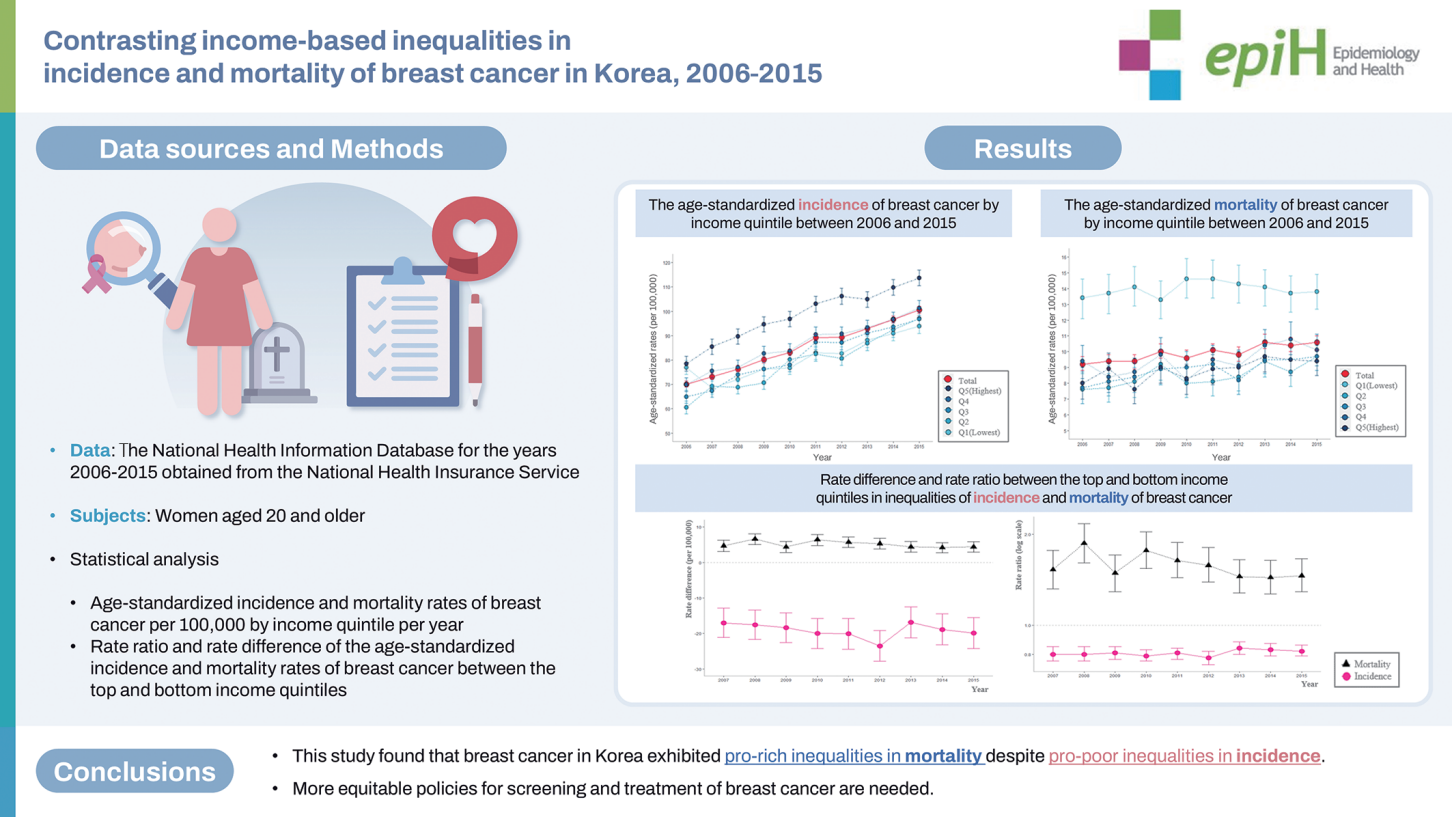


**Table 1.** Rate difference (RD) and rate ratio (RR) between the top and bottom income quintiles, and p-values for trends in inequalities of incidence and mortality of breast cancer in 2006-2015 in Korea^1^

Year	Incidence		Mortality	
	RD (95% CI)	RR (95% CI)	RD (95% CI)	RR (95% CI)
2006	-1.5 (-5.7, 2.7)	0.98 (0.93, 1.04)	5.4 (3.8, 7.0)	1.67 (1.44, 1.95)
2007	-17.0 (-21.1, -12.9)	0.80 (0.76, 0.85)	4.7 (3.1, 6.3)	1.53 (1.32, 1.77)
2008	-17.6 (-21.7, -13.4)	0.80 (0.76, 0.85)	6.6 (5.0, 8.1)	1.87 (1.61, 2.17)
2009	-18.4 (-22.6, -14.2)	0.81 (0.77, 0.85)	4.4 (2.8, 5.9)	1.49 (1.29, 1.71)
2010	-20.0 (-24.3, -15.8)	0.79 (0.76, 0.83)	6.4 (4.8, 7.9)	1.77 (1.54, 2.04)
2011	-20.1 (-24.4, -15.8)	0.81 (0.77, 0.84)	5.7 (4.2, 7.2)	1.64 (1.44, 1.88)
2012	-23.5 (-27.8, -19.2)	0.78 (0.74, 0.82)	5.3 (3.8, 6.8)	1.58 (1.39, 1.81)
2013	-16.9 (-21.2, -12.5)	0.84 (0.80, 0.88)	4.4 (2.9, 5.9)	1.45 (1.28, 1.65)
2014	-18.9 (-23.2, -14.5)	0.83 (0.79, 0.87)	4.2 (2.7, 5.6)	1.44 (1.27, 1.64)
2015	-19.9 (-24.3, -15.5)	0.82 (0.79, 0.86)	4.4 (2.9, 5.8)	1.46 (1.29, 1.66)
p for trend 2006-2015	0.071	0.352	0.168	0.087
p for trend 2007-2015	0.336	0.132	0.178	0.122

CI, confidence interval.

1 The top quintile group was used as the reference group.

**Table 2.** Age group–specific rate differences (RDs) and rate ratios (RRs) in the incidence and mortality of breast cancer in 2015 in Korea

	Age group-specific rate			RD (95% CI)^1^	RR (95% CI)^1^
	Total	Q1 (lowest)	Q5 (highest)		
Incidence					
20-24	2.1 (1.4-2.8)	2.2 (0.6-3.8)	2.5 (0.8-4.2)	-0.3 (-2.7, 2.0)	0.87 (0.32, 2.41)
25-29	12.3 (10.5-14.0)	11.7 (7.8-15.6)	10.7 (7.0-14.4)	1.0 (-4.4, 6.3)	1.09 (0.67, 1.76)
30-34	34.3 (31.6-36.9)	30.0 (24.5-35.6)	33.8 (27.9-39.7)	-3.8 (-11.9, 4.3)	0.89 (0.69, 1.15)
35-39	79.9 (75.8-83.9)	73.4 (64.7-82.1)	90.0 (80.4-99.6)	-16.6 (-29.6, -3.6)	0.82 (0.70, 0.96)
40-44	148.7 (143.5-153.8)	128.2 (117.6-138.8)	176.0 (163.5-188.4)	-47.8 (-64.1, -31.4)	0.73 (0.65, 0.81)
45-49	191.3 (185.4-197.2)	172.1 (159.6-184.6)	207.6 (193.9-221.3)	-35.5 (-54.0, -16.9)	0.83 (0.75, 0.91)
50-54	161.8 (156.3-167.2)	142.9 (131.5-154.3)	193.4 (180.2-206.7)	-50.6 (-68.1, -33.0)	0.74 (0.66, 0.82)
55-59	152.6 (147.0-158.2)	149.7 (137.4-162.1)	158.1 (145.3-170.8)	-8.3 (-26.1, 9.4)	0.95 (0.84, 1.06)
60-64	134.0 (127.7-140.3)	138.4 (124.1-152.7)	159.2 (143.9-174.5)	-20.8 (-41.7, 0.2)	0.87 (0.76, 1.00)
65-69	126.5 (119.7-133.3)	119.9 (105.1-134.6)	150.2 (133.7-166.7)	-30.3 (-52.5, -8.2)	0.80 (0.68, 0.94)
70-74	96.6 (90.4-102.7)	105.1 (90.8-119.5)	109.5 (94.8-124.1)	-4.3 (-24.9, 16.2)	0.96 (0.79, 1.16)
75-79	68.5 (62.8-74.3)	83.5 (69.3-97.8)	78.7 (64.9-92.6)	4.8 (-15.1, 24.7)	1.06 (0.83, 1.36)
80-84	47.2 (41.2-53.3)	53.2 (39.0-67.4)	58.1 (43.1-73.0)	-4.9 (-25.5, 15.7)	0.92 (0.63, 1.33)
≥85	30.9 (25.2-36.7)	27.5 (15.7-39.2)	37.0 (23.0-50.9)	-9.5 (-27.7, 8.7)	0.74 (0.42, 1.31)
Mortality					
20-24	0.1 (0.0-0.2)	0.3 (0.0-0.9)	0.0 (0.0-0.0)	0.3 (-0.3, 0.9)	N/A
25-29	0.6 (0.2-1.0)	1.0 (0.0-2.1)	1.3 (0.0-2.7)	-0.3 (-2.1, 1.4)	0.75 (0.17, 3.34)
30-34	2.1 (1.5-2.8)	3.2 (1.4-5.0)	1.1 (0.0-2.1)	2.1 (0.0, 4.2)	3.00 (0.97, 9.29)
35-39	5.4 (4.3-6.4)	6.7 (4.1-9.3)	3.2 (1.4-5.0)	3.5 (0.3, 6.7)	2.08 (1.04, 4.14)
40-44	9.0 (7.8-10.3)	11.4 (8.2-14.5)	8.3 (5.6-11.0)	3.1 (-1.1, 7.2)	1.37 (0.89, 2.10)
45-49	14.3 (12.7-15.9)	18.3 (14.2-22.4)	14.1 (10.5-17.7)	4.2 (-1.2, 9.6)	1.30 (0.93, 1.82)
50-54	16.4 (14.6-18.1)	23.0 (18.4-27.6)	9.5 (6.6-12.4)	13.5 (8.1, 19.0)	2.42 (1.68, 3.50)
55-59	18.8 (16.8-20.7)	26.3 (21.1-31.5)	15.2 (11.2-19.1)	11.2 (4.7, 17.7)	1.74 (1.25, 2.40)
60-64	17.8 (15.5-20.1)	22.3 (16.6-28.0)	16.1 (11.3-21.0)	6.2 (-1.4, 13.7)	1.38 (0.93, 2.05)
65-69	17.3 (14.8-19.9)	21.3 (15.1-27.6)	20.4 (14.3-26.5)	0.9 (-7.8, 9.7)	1.05 (0.69, 1.59)
70-74	18.0 (15.3-20.7)	25.0 (18.0-32.0)	17.9 (12.0-23.8)	7.1 (-2.1, 16.3)	1.40 (0.91, 2.16)
75-79	17.4 (14.5-20.3)	20.9 (13.8-28.0)	16.5 (10.2-22.8)	4.4 (-5.2, 13.9)	1.27 (0.76, 2.12)
80-84	21.2 (17.2-25.3)	27.6 (17.4-37.8)	26.0 (16.0-36.0)	1.5 (-12.8, 15.8)	1.06 (0.62, 1.81)
≥85	26.8 (21.5-32.2)	26.2 (14.7-37.6)	32.9 (19.7-46.0)	-6.7 (-24.2, 10.7)	0.80 (0.44, 1.44)

CI, confidence interval; N/A, not avilable.

1 The top quintile group was used as the reference group.

## Supplementary materials

Supplementary Material 1. Annual numbers of population and associated numbers of incidence and deaths of breast cancer according to income quintiles between 2006 and 2015 in Korea

Supplementary Material 2. Rate difference in incidence and mortality of breast cancer in 2006-2015 (Reference group: Q5, the highest income group)

Supplementary Material 3. Rate ratios in incidence and mortality of breast cancer in 2006-2015 (Reference group: Q5, the highest income group)

Supplementary Material 4. The incidence and mortality rates of breast cancer by age groups and income quintile in 2006

Supplementary Material 5. The incidence and mortality rates of breast cancer by age groups and income quintile in 2010

Supplementary Material 6. The incidence and mortality rates of breast cancer by age groups and income quintile in 2015

Supplementary Material 7. The age-standardized incidence, mortality rates, and mortality-to-incidence ratios of breast cancer by income quintile between 2006 and 2015 in Korea
