# Supplementary material for: Contrasting income-based inequalities in incidence and mortality of breast cancer in Korea, 2006-2015
Source: Epidemiol Health. 2024 Sep 11;46:e2024074. doi: 10.4178/epih.e2024074 (PMC11826041; doi:10.4178/epih.e2024074)
Supplement: Supplementary Material 1. — Annual numbers of population and associated numbers of incidence and deaths of breast cancer according to income quintiles between 2006 and 2015 in Korea [file epih-46-e2024074-Supplementary-1.docx]

Supplementary Material 1. Annual numbers of population and associated numbers of incidence and deaths of breast cancer according to income quintiles between 2006 and 2015 in Korea

|  | Q1 (lowest) | | | Q2 | | | Q3 | | | Q4 | | | Q5 (highest) | | | |
| --- | --- | --- | --- | --- | --- | --- | --- | --- | --- | --- | --- | --- | --- | --- | --- | --- |
| Year | No. of population | No. of incident case | No. of deaths | No. of population | No. of incident case | No. of deaths | No. of population | No. of incident case | No. of deaths | No. of population | No. of incident case | No. of deaths | No. of population | No. of incident case | No. of deaths |  |
| 2006 | 3,648,039 | 2,648 | 450 | 3,594,365 | 2,097 | 250 | 3,612,387 | 2,243 | 254 | 3,588,213 | 2,411 | 314 | 3,564,921 | 2,671 | 266 |  |
| 2007 | 3,667,551 | 2,433 | 472 | 3,630,970 | 2,448 | 262 | 3,640,474 | 2,378 | 278 | 3,643,454 | 2,664 | 285 | 3,598,548 | 3,012 | 304 |  |
| 2008 | 3,720,405 | 2,622 | 505 | 3,693,972 | 2,491 | 282 | 3,693,319 | 2,687 | 296 | 3,699,075 | 2,809 | 303 | 3,701,195 | 3,256 | 266 |  |
| 2009 | 3,774,914 | 2,844 | 490 | 3,713,220 | 2,613 | 330 | 3,744,934 | 2,834 | 324 | 3,741,672 | 3,068 | 360 | 3,743,140 | 3,513 | 326 |  |
| 2010 | 3,726,878 | 2,862 | 545 | 3,711,962 | 2,989 | 294 | 3,718,051 | 2,910 | 334 | 3,715,579 | 3,123 | 304 | 3,717,386 | 3,614 | 305 |  |
| 2011 | 3,851,742 | 3,214 | 569 | 3,798,738 | 3,175 | 310 | 3,818,573 | 3,366 | 357 | 3,822,917 | 3,492 | 366 | 3,822,476 | 3,974 | 343 |  |
| 2012 | 3,882,889 | 3,255 | 571 | 3,849,796 | 3,151 | 332 | 3,864,125 | 3,411 | 326 | 3,865,611 | 3,551 | 361 | 3,863,874 | 4,159 | 358 |  |
| 2013 | 3,923,293 | 3,518 | 577 | 3,897,121 | 3,457 | 377 | 3,905,773 | 3,627 | 385 | 3,906,893 | 3,706 | 421 | 3,907,923 | 4,176 | 393 |  |
| 2014 | 3,952,397 | 3,691 | 568 | 3,942,692 | 3,719 | 362 | 3,935,958 | 3,771 | 395 | 3,943,633 | 3,904 | 449 | 3,943,260 | 4,431 | 394 |  |
| 2015 | 4,034,291 | 3,915 | 598 | 4,005,349 | 4,006 | 418 | 4,018,386 | 4,004 | 420 | 4,019,189 | 4,184 | 433 | 4,018,721 | 4,709 | 409 |  |
