# Supplementary material for: Contrasting income-based inequalities in incidence and mortality of breast cancer in Korea, 2006-2015
Source: Epidemiol Health. 2024 Sep 11;46:e2024074. doi: 10.4178/epih.e2024074 (PMC11826041; doi:10.4178/epih.e2024074)
Supplement: Supplementary Material 2. — Rate difference in incidence and mortality of breast cancer in 2006-2015 (Reference group: Q5, the highest income group) [file epih-46-e2024074-Supplementary-2.docx]

Supplementary Material 2. Rate difference in incidence and mortality of breast cancer in 2006-2015 (Reference group: Q5, the highest income group)

|  | Q1 (lowest) | Q2 | Q3 | Q4 |
| --- | --- | --- | --- | --- |
| Incidence | | | | |
| 2006 | -1.5 (-5.7 to 2.7) | -18.0 (-21.9 to -14.0) | -13.6 (-17.6 to -9.5) | -8.7 (-12.8 to -4.6) |
| 2007 | -17.0 (-21.1 to -12.9) | -16.3 (-20.4 to -12.2) | -18.1 (-22.2 to -14.0) | -10.0 (-14.3 to -5.8) |
| 2008 | -17.6 (-21.7 to -13.4) | -20.8 (-25.0 to -16.7) | -15.8 (-20.0 to -11.6) | -12.6 (-16.8 to -8.3) |
| 2009 | -18.4 (-22.6 to -14.2) | -24.0 (-28.2 to -19.9) | -18.5 (-22.7 to -14.3) | -12.0 (-16.3 to -7.7) |
| 2010 | -20.0 (-24.3 to -15.8) | -16.8 (-21.0 to -12.5) | -18.9 (-23.2 to -14.7) | -13.1 (-17.4 to -8.8) |
| 2011 | -20.1 (-24.4 to -15.8) | -20.5 (-24.8 to -16.2) | -15.7 (-20.0 to -11.3) | -12.5 (-16.9 to -8.1) |
| 2012 | -23.5 (-27.8 to -19.2) | -25.5 (-29.8 to -21.2) | -19.0 (-23.4 to -14.7) | -15.5 (-19.9 to -11.1) |
| 2013 | -16.9 (-21.2 to -12.5) | -18.1 (-22.4 to -13.8) | -13.8 (-18.1 to -9.4) | -11.6 (-16.0 to -7.2) |
| 2014 | -18.9 (-23.2 to -14.5) | -17.4 (-21.8 to -13.0) | -16.2 (-20.6 to -11.7) | -13.1 (-17.6 to -8.7) |
| 2015 | -19.9 (-24.3 to -15.5) | -16.8 (-21.2 to -12.3) | -16.9 (-21.3 to -12.4) | -12.4 (-16.9 to -7.9) |
| Mortality | | | | |
| 2006 | 5.4 (3.8 to 7.0) | -0.4 (-1.7 to 1.0) | -0.3 (-1.7 to 1.1) | 1.4 (-0.1 to 2.8) |
| 2007 | 4.7 (3.1 to 6.3) | -1.2 (-2.6 to 0.1) | -0.8 (-2.2 to 0.6) | -0.6 (-2.0 to 0.8) |
| 2008 | 6.6 (5.0 to 8.1) | 0.5 (-0.8 to 1.8) | 0.8 (-0.5 to 2.1) | 1.1 (-0.2 to 2.4) |
| 2009 | 4.4 (2.8 to 5.9) | 0.2 (-1.2 to 1.6) | 0.0 (-1.4 to 1.3) | 0.9 (-0.5 to 2.3) |
| 2010 | 6.4 (4.8 to 7.9) | -0.3 (-1.6 to 1.0) | 0.8 (-0.6 to 2.1) | 0.0 (-1.3 to 1.3) |
| 2011 | 5.7 (4.2 to 7.2) | -0.8 (-2.1 to 0.5) | 0.4 (-1.0 to 1.7) | 0.6 (-0.8 to 1.9) |
| 2012 | 5.3 (3.8 to 6.8) | -0.6 (-1.9 to 0.7) | -0.8 (-2.1 to 0.5) | 0.1 (-1.2 to 1.4) |
| 2013 | 4.4 (2.9 to 5.9) | -0.3 (-1.7 to 1.0) | -0.2 (-1.5 to 1.2) | 0.7 (-0.7 to 2.1) |
| 2014 | 4.2 (2.7 to 5.6) | -0.8 (-2.1 to 0.5) | 0.0 (-1.3 to 1.3) | 1.4 (0.0 to 2.8) |
| 2015 | 4.4 (2.9 to 5.8) | 0.2 (-1.1 to 1.6) | 0.3 (-1.0 to 1.6) | 0.7 (-0.7 to 2.0) |
