# Supplementary material for: Contrasting income-based inequalities in incidence and mortality of breast cancer in Korea, 2006-2015
Source: Epidemiol Health. 2024 Sep 11;46:e2024074. doi: 10.4178/epih.e2024074 (PMC11826041; doi:10.4178/epih.e2024074)
Supplement: Supplementary Material 3. — Rate ratios in incidence and mortality of breast cancer in 2006-2015 (Reference group: Q5, the highest income group) [file epih-46-e2024074-Supplementary-3.docx]

Supplementary Material 3. Rate ratios in incidence and mortality of breast cancer in 2006-2015 (Reference group: Q5, the highest income group)

|  | Q1 (lowest) | Q2 | Q3 | Q4 |
| --- | --- | --- | --- | --- |
| Incidence | | | | |
| 2006 | 0.98 (0.93 - 1.04) | 0.77 (0.73 - 0.82) | 0.83 (0.78 - 0.88) | 0.89 (0.84 - 0.94) |
| 2007 | 0.80 (0.76 - 0.85) | 0.81 (0.77 - 0.85) | 0.79 (0.75 - 0.83) | 0.88 (0.84 - 0.93) |
| 2008 | 0.80 (0.76 - 0.85) | 0.77 (0.73 - 0.81) | 0.82 (0.78 - 0.87) | 0.86 (0.82 - 0.90) |
| 2009 | 0.81 (0.77 - 0.85) | 0.75 (0.71 - 0.79) | 0.80 (0.77 - 0.85) | 0.87 (0.83 - 0.92) |
| 2010 | 0.79 (0.76 - 0.83) | 0.83 (0.79 - 0.87) | 0.80 (0.77 - 0.84) | 0.86 (0.82 - 0.91) |
| 2011 | 0.81 (0.77 - 0.84) | 0.80 (0.76 - 0.84) | 0.85 (0.81 - 0.89) | 0.88 (0.84 - 0.92) |
| 2012 | 0.78 (0.74 - 0.82) | 0.76 (0.72 - 0.80) | 0.82 (0.78 - 0.86) | 0.85 (0.82 - 0.89) |
| 2013 | 0.84 (0.80 - 0.88) | 0.83 (0.79 - 0.87) | 0.87 (0.83 - 0.91) | 0.89 (0.85 - 0.93) |
| 2014 | 0.83 (0.79 - 0.87) | 0.84 (0.81 - 0.88) | 0.85 (0.82 - 0.89) | 0.88 (0.84 - 0.92) |
| 2015 | 0.82 (0.79 - 0.86) | 0.85 (0.82 - 0.89) | 0.85 (0.82 - 0.89) | 0.89 (0.85 - 0.93) |
| Mortality | | | | |
| 2006 | 1.67 (1.44 - 1.95) | 0.95 (0.80 - 1.14) | 0.96 (0.81 - 1.14) | 1.17 (0.99 - 1.38) |
| 2007 | 1.53 (1.32 - 1.77) | 0.86 (0.73 - 1.02) | 0.91 (0.77 - 1.07) | 0.94 (0.80 - 1.10) |
| 2008 | 1.87 (1.61 - 2.17) | 1.07 (0.90 - 1.26) | 1.11 (0.94 - 1.31) | 1.14 (0.97 - 1.35) |
| 2009 | 1.49 (1.29 - 1.71) | 1.02 (0.88 - 1.19) | 0.99 (0.85 - 1.16) | 1.10 (0.95 - 1.28) |
| 2010 | 1.77 (1.54 - 2.04) | 0.96 (0.82 - 1.13) | 1.09 (0.94 - 1.28) | 1.00 (0.85 - 1.17) |
| 2011 | 1.64 (1.44 - 1.88) | 0.91 (0.78 - 1.07) | 1.04 (0.90 - 1.21) | 1.07 (0.92 - 1.23) |
| 2012 | 1.58 (1.39 - 1.81) | 0.93 (0.80 - 1.08) | 0.91 (0.78 - 1.06) | 1.01 (0.87 - 1.17) |
| 2013 | 1.45 (1.28 - 1.65) | 0.96 (0.84 - 1.11) | 0.98 (0.85 - 1.13) | 1.07 (0.93 - 1.23) |
| 2014 | 1.44 (1.27 - 1.64) | 0.92 (0.80 - 1.06) | 1.00 (0.87 - 1.15) | 1.15 (1.00 - 1.31) |
| 2015 | 1.46 (1.29 - 1.66) | 1.03 (0.89 - 1.18) | 1.03 (0.90 - 1.18) | 1.07 (0.93 - 1.23) |
