# Supplementary material for: Contrasting income-based inequalities in incidence and mortality of breast cancer in Korea, 2006-2015
Source: Epidemiol Health. 2024 Sep 11;46:e2024074. doi: 10.4178/epih.e2024074 (PMC11826041; doi:10.4178/epih.e2024074)
Supplement: Supplementary Material 4. — The incidence and mortality rates of breast cancer by age groups and income quintile in 2006 [file epih-46-e2024074-Supplementary-4.docx]

Supplementary Material 4. The incidence and mortality rates of breast cancer by age groups and income quintile in 2006

|  | Total | Q1 (lowest) | Q2 | Q3 | Q4 | Q5 (highest) |
| --- | --- | --- | --- | --- | --- | --- |
| Incidence |  |  |  |  |  |  |
| 20-24 | 1.6 (1.0 - 2.2) | 0.8 (0.0 - 1.8) | 0.6 (0.0 - 1.4) | 2.0 (0.5 - 3.5) | 1.7 (0.3 - 3.0) | 3.2 (1.3 - 5.1) |
| 25-29 | 10.0 (8.5 - 11.4) | 10.5 (7.3 - 13.8) | 10.5 (7.2 - 13.8) | 10.8 (7.6 - 14.1) | 8.8 (5.7 - 11.9) | 9.1 (6.0 - 12.1) |
| 30-34 | 27.1 (24.9 - 29.4) | 22.9 (18.3 - 27.5) | 26.0 (21.1 - 30.9) | 29.6 (24.3 - 34.8) | 29.6 (24.4 - 34.9) | 27.6 (22.6 - 32.7) |
| 35-39 | 56.8 (53.6 - 60.0) | 52.1 (45.2 - 58.9) | 54.6 (47.6 - 61.6) | 56.9 (49.7 - 64.2) | 56.7 (49.5 - 63.8) | 64.0 (56.3 - 71.6) |
| 40-44 | 105.6 (101.1 - 110.0) | 103.5 (93.7 - 113.3) | 95.9 (86.5 - 105.4) | 98.3 (88.7 - 107.9) | 112.8 (102.6 - 123.0) | 117.5 (106.9 - 128.1) |
| 45-49 | 134.6 (129.4 - 139.7) | 137.0 (125.4 - 148.5) | 119.2 (108.4 - 130.0) | 129.5 (118.3 - 140.8) | 134.6 (123.1 - 146.2) | 152.6 (140.3 - 164.8) |
| 50-54 | 116.6 (111.0 - 122.1) | 122.4 (109.7 - 135.1) | 99.9 (88.4 - 111.4) | 109.5 (97.4 - 121.6) | 116.8 (104.3 - 129.2) | 134.4 (121.0 - 147.8) |
| 55-59 | 104.3 (98.3 - 110.2) | 113.2 (99.4 - 127.0) | 85.9 (73.9 - 98.0) | 98.8 (85.9 - 111.7) | 110.0 (96.4 - 123.6) | 113.4 (99.5 - 127.3) |
| 60-64 | 95.2 (89.2 - 101.1) | 123.3 (108.2 - 138.5) | 82.4 (69.9 - 94.8) | 77.5 (65.4 - 89.6) | 89.9 (77.0 - 102.9) | 102.5 (88.5 - 116.4) |
| 65-69 | 76.7 (71.0 - 82.4) | 100.6 (86.1 - 115.1) | 54.4 (43.7 - 65.1) | 71.2 (59.2 - 83.3) | 71.6 (59.2 - 84.1) | 85.8 (72.3 - 99.3) |
| 70-74 | 60.7 (54.9 - 66.5) | 100.0 (83.6 - 116.5) | 41.0 (30.5 - 51.6) | 39.9 (29.4 - 50.3) | 46.7 (35.3 - 58.0) | 75.6 (61.1 - 90.0) |
| 75-79 | 41.0 (35.3 - 46.8) | 68.6 (53.1 - 84.2) | 29.3 (17.6 - 41.0) | 27.1 (16.7 - 37.5) | 29.9 (19.0 - 40.7) | 44.9 (31.3 - 58.5) |
| 80-84 | 31.1 (24.6 - 37.6) | 63.6 (42.8 - 84.3) | 15.9 (5.5 - 26.3) | 15.8 (5.5 - 26.1) | 21.3 (9.3 - 33.4) | 39.0 (22.7 - 55.2) |
| 85+ | 17.4 (11.2 - 23.5) | 35.8 (16.4 - 55.3) | 16.9 (3.4 - 30.4) | 8.3 (0.0 - 17.8) | 17.0 (3.4 - 30.6) | 8.4 (0.0 - 17.9) |
| Mortality |  |  |  |  |  |  |
| 20-24 | 0.3 (0.0 - 0.5) | 0.0 (0.0 - 0.0) | 0.0 (0.0 - 0.0) | 0.3 (0.0 - 0.8) | 0.6 (0.0 - 1.3) | 0.6 (0.0 - 1.4) |
| 25-29 | 0.2 (0.0 - 0.3) | 0.3 (0.0 - 0.8) | 0.0 (0.0 - 0.0) | 0.3 (0.0 - 0.7) | 0.3 (0.0 - 0.8) | 0.0 (0.0 - 0.0) |
| 30-34 | 2.6 (1.9 - 3.3) | 4.3 (2.3 - 6.2) | 1.4 (0.3 - 2.6) | 1.9 (0.6 - 3.3) | 3.3 (1.6 - 5.1) | 2.2 (0.8 - 3.6) |
| 35-39 | 5.7 (4.7 - 6.7) | 8.7 (5.9 - 11.5) | 4.9 (2.8 - 7.0) | 5.0 (2.9 - 7.2) | 4.4 (2.4 - 6.4) | 5.5 (3.2 - 7.7) |
| 40-44 | 9.2 (7.9 - 10.5) | 15.3 (11.5 - 19.0) | 5.1 (2.9 - 7.3) | 6.1 (3.7 - 8.6) | 11.2 (8.0 - 14.4) | 8.0 (5.2 - 10.8) |
| 45-49 | 12.5 (10.9 - 14.0) | 19.5 (15.1 - 23.9) | 9.9 (6.8 - 13.0) | 9.7 (6.6 - 12.7) | 14.4 (10.6 - 18.2) | 8.9 (6.0 - 11.9) |
| 50-54 | 14.0 (12.1 - 15.9) | 24.4 (18.7 - 30.1) | 11.4 (7.5 - 15.4) | 14.3 (9.9 - 18.7) | 9.7 (6.1 - 13.3) | 10.1 (6.4 - 13.8) |
| 55-59 | 16.9 (14.5 - 19.3) | 20.6 (14.7 - 26.5) | 19.4 (13.7 - 25.1) | 14.1 (9.2 - 18.9) | 21.8 (15.8 - 27.9) | 8.5 (4.7 - 12.3) |
| 60-64 | 14.1 (11.8 - 16.4) | 14.6 (9.4 - 19.8) | 11.2 (6.6 - 15.8) | 7.9 (4.0 - 11.8) | 13.6 (8.6 - 18.7) | 23.3 (16.6 - 29.9) |
| 65-69 | 13.9 (11.5 - 16.4) | 17.5 (11.4 - 23.6) | 12.1 (7.0 - 17.1) | 13.8 (8.5 - 19.1) | 10.7 (5.9 - 15.5) | 15.5 (9.8 - 21.2) |
| 70-74 | 12.7 (10.0 - 15.3) | 19.7 (12.4 - 27.0) | 12.0 (6.3 - 17.7) | 15.0 (8.6 - 21.4) | 10.1 (4.8 - 15.3) | 6.5 (2.2 - 10.7) |
| 75-79 | 14.2 (10.8 - 17.6) | 15.6 (8.2 - 23.0) | 9.8 (3.0 - 16.5) | 9.4 (3.3 - 15.5) | 21.6 (12.4 - 30.9) | 13.9 (6.3 - 21.5) |
| 80-84 | 17.7 (12.8 - 22.6) | 22.9 (10.5 - 35.4) | 19.5 (8.0 - 31.0) | 14.1 (4.3 - 23.8) | 14.2 (4.4 - 24.1) | 17.7 (6.7 - 28.7) |
| 85+ | 25.2 (17.8 - 32.5) | 44.1 (22.5 - 65.7) | 14.1 (1.7 - 26.4) | 19.5 (5.0 - 33.9) | 19.8 (5.1 - 34.6) | 28.0 (10.7 - 45.4) |
