# Supplementary material for: Contrasting income-based inequalities in incidence and mortality of breast cancer in Korea, 2006-2015
Source: Epidemiol Health. 2024 Sep 11;46:e2024074. doi: 10.4178/epih.e2024074 (PMC11826041; doi:10.4178/epih.e2024074)
Supplement: Supplementary Material 5. — The incidence and mortality rates of breast cancer by age groups and income quintile in 2010 [file epih-46-e2024074-Supplementary-5.docx]

Supplementary Material 5. The incidence and mortality rates of breast cancer by age groups and income quintile in 2010

|  | Total | Q1 (lowest) | Q2 | Q3 | Q4 | Q5 (highest) |
| --- | --- | --- | --- | --- | --- | --- |
| Incidence |  |  |  |  |  |  |
| 20-24 | 2.7 (1.9 - 3.6) | 3.8 (1.6 - 6.1) | 4.2 (1.8 - 6.5) | 1.0 (0.0 - 2.2) | 1.4 (0.0 - 2.8) | 3.1 (1.1 - 5.2) |
| 25-29 | 10.9 (9.4 - 12.4) | 9.5 (6.4 - 12.7) | 10.5 (7.2 - 13.9) | 10.4 (7.1 - 13.7) | 11.3 (7.9 - 14.8) | 12.9 (9.2 - 16.6) |
| 30-34 | 31.6 (29.0 - 34.2) | 31.5 (25.8 - 37.3) | 33.0 (27.1 - 38.9) | 29.8 (24.2 - 35.4) | 31.8 (26.0 - 37.6) | 31.8 (26.0 - 37.6) |
| 35-39 | 71.6 (68.0 - 75.2) | 61.4 (54.0 - 68.9) | 70.4 (62.4 - 78.4) | 72.5 (64.4 - 80.7) | 70.6 (62.6 - 78.6) | 83.2 (74.5 - 91.8) |
| 40-44 | 118.0 (113.3 - 122.7) | 109.6 (99.5 - 119.6) | 109.5 (99.4 - 119.5) | 110.2 (100.1 - 120.3) | 121.6 (111.0 - 132.2) | 139.3 (127.9 - 150.6) |
| 45-49 | 159.9 (154.4 - 165.3) | 142.2 (130.7 - 153.7) | 149.9 (138.1 - 161.7) | 150.2 (138.4 - 162.0) | 163.0 (150.7 - 175.3) | 194.2 (180.8 - 207.6) |
| 50-54 | 141.5 (136.1 - 146.9) | 126.8 (115.3 - 138.3) | 147.6 (135.2 - 159.9) | 135.4 (123.6 - 147.3) | 144.0 (131.8 - 156.2) | 153.6 (140.9 - 166.2) |
| 55-59 | 121.5 (115.5 - 127.5) | 127.6 (113.8 - 141.4) | 110.6 (97.8 - 123.4) | 104.4 (91.9 - 116.8) | 123.3 (109.8 - 136.9) | 141.5 (127.0 - 156.0) |
| 60-64 | 114.9 (108.5 - 121.4) | 108.0 (94.1 - 122.0) | 108.5 (94.5 - 122.6) | 111.4 (97.2 - 125.6) | 116.5 (102.0 - 131.1) | 130.3 (114.9 - 145.6) |
| 65-69 | 95.3 (89.3 - 101.4) | 89.9 (76.8 - 102.9) | 93.2 (79.9 - 106.6) | 94.0 (80.6 - 107.5) | 81.1 (68.6 - 93.5) | 118.4 (103.4 - 133.5) |
| 70-74 | 68.2 (62.6 - 73.8) | 62.1 (50.2 - 73.9) | 67.4 (55.0 - 79.7) | 52.6 (41.7 - 63.5) | 75.7 (62.6 - 88.8) | 83.3 (69.6 - 97.1) |
| 75-79 | 49.4 (43.8 - 55.1) | 45.2 (33.1 - 57.3) | 41.9 (30.2 - 53.7) | 46.6 (34.3 - 58.9) | 52.5 (39.4 - 65.5) | 60.9 (46.9 - 75.0) |
| 80-84 | 33.8 (27.8 - 39.8) | 27.9 (15.7 - 40.1) | 33.5 (20.1 - 47.0) | 32.1 (19.0 - 45.2) | 25.1 (13.5 - 36.7) | 50.3 (33.8 - 66.7) |
| 85+ | 21.7 (15.9 - 27.5) | 17.3 (6.0 - 28.7) | 17.5 (5.4 - 29.6) | 24.6 (10.7 - 38.5) | 26.6 (12.1 - 41.1) | 22.5 (9.2 - 35.8) |
| Mortality |  |  |  |  |  |  |
| 20-24 | 0.1 (0.0 - 0.3) | 0.3 (0.0 - 1.0) | 0.0 (0.0 - 0.0) | 0.0 (0.0 - 0.0) | 0.0 (0.0 - 0.0) | 0.3 (0.0 - 1.0) |
| 25-29 | 0.7 (0.3 - 1.1) | 1.9 (0.5 - 3.3) | 0.3 (0.0 - 0.8) | 0.8 (0.0 - 1.7) | 0.3 (0.0 - 0.8) | 0.3 (0.0 - 0.8) |
| 30-34 | 1.9 (1.3 - 2.6) | 3.6 (1.6 - 5.5) | 2.2 (0.7 - 3.7) | 0.6 (0.0 - 1.3) | 1.9 (0.5 - 3.3) | 1.4 (0.2 - 2.6) |
| 35-39 | 5.5 (4.5 - 6.5) | 9.4 (6.5 - 12.4) | 3.1 (1.4 - 4.7) | 4.5 (2.5 - 6.5) | 4.0 (2.1 - 5.9) | 6.4 (4.0 - 8.8) |
| 40-44 | 7.9 (6.7 - 9.1) | 12.0 (8.7 - 15.4) | 5.1 (2.9 - 7.2) | 6.7 (4.2 - 9.2) | 9.6 (6.6 - 12.6) | 6.0 (3.7 - 8.4) |
| 45-49 | 13.7 (12.1 - 15.3) | 23.7 (19.0 - 28.4) | 11.1 (7.9 - 14.3) | 13.5 (10.0 - 17.1) | 11.1 (7.9 - 14.3) | 8.9 (6.1 - 11.8) |
| 50-54 | 16.2 (14.4 - 18.0) | 24.8 (19.7 - 29.9) | 12.4 (8.8 - 16.0) | 17.5 (13.3 - 21.8) | 13.8 (10.0 - 17.5) | 12.4 (8.8 - 16.0) |
| 55-59 | 16.3 (14.1 - 18.5) | 20.5 (15.0 - 26.0) | 13.1 (8.7 - 17.6) | 16.2 (11.3 - 21.1) | 15.5 (10.7 - 20.3) | 16.2 (11.3 - 21.2) |
| 60-64 | 17.2 (14.7 - 19.7) | 21.2 (15.0 - 27.4) | 16.0 (10.7 - 21.4) | 17.0 (11.4 - 22.5) | 15.1 (9.9 - 20.3) | 16.5 (11.0 - 22.0) |
| 65-69 | 13.4 (11.2 - 15.7) | 19.4 (13.3 - 25.4) | 13.5 (8.4 - 18.5) | 10.4 (6.0 - 14.9) | 9.5 (5.2 - 13.7) | 14.4 (9.2 - 19.7) |
| 70-74 | 15.8 (13.2 - 18.5) | 22.5 (15.3 - 29.6) | 18.3 (11.9 - 24.8) | 14.8 (9.0 - 20.6) | 12.4 (7.1 - 17.7) | 11.2 (6.2 - 16.3) |
| 75-79 | 16.9 (13.6 - 20.2) | 23.4 (14.8 - 32.1) | 15.4 (8.3 - 22.5) | 17.8 (10.2 - 25.4) | 16.1 (8.8 - 23.3) | 11.8 (5.6 - 18.1) |
| 80-84 | 19.0 (14.5 - 23.5) | 33.5 (20.1 - 46.9) | 11.2 (3.4 - 18.9) | 15.4 (6.3 - 24.4) | 9.8 (2.5 - 17.0) | 25.1 (13.5 - 36.7) |
| 85+ | 16.0 (11.0 - 21.0) | 32.7 (17.2 - 48.3) | 15.3 (4.0 - 26.6) | 10.2 (1.3 - 19.2) | 8.2 (0.2 - 16.2) | 12.3 (2.5 - 22.1) |
