# Supplementary material for: Contrasting income-based inequalities in incidence and mortality of breast cancer in Korea, 2006-2015
Source: Epidemiol Health. 2024 Sep 11;46:e2024074. doi: 10.4178/epih.e2024074 (PMC11826041; doi:10.4178/epih.e2024074)
Supplement: Supplementary Material 6. — The incidence and mortality rates of breast cancer by age groups and income quintile in 2015 [file epih-46-e2024074-Supplementary-6.docx]

Supplementary Material 6. The incidence and mortality rates of breast cancer by age groups and income quintile in 2015

|  | Total | Q1 (lowest) | Q2 | Q3 | Q4 | Q5 (highest) |
| --- | --- | --- | --- | --- | --- | --- |
| Incidence |  |  |  |  |  |  |
| 20-24 | 2.1 (1.4 - 2.8) | 2.2 (0.6 - 3.8) | 0.9 (0.0 - 2.0) | 2.5 (0.8 - 4.2) | 2.5 (0.8 - 4.2) | 2.5 (0.8 - 4.2) |
| 25-29 | 12.3 (10.5 - 14.0) | 11.7 (7.8 - 15.6) | 15.1 (10.7 - 19.6) | 11.1 (7.3 - 14.8) | 12.7 (8.7 - 16.8) | 10.7 (7.0 - 14.4) |
| 30-34 | 34.3 (31.6 - 36.9) | 30.0 (24.5 - 35.6) | 30.3 (24.7 - 35.9) | 36.0 (29.9 - 42.1) | 41.1 (34.6 - 47.6) | 33.8 (27.9 - 39.7) |
| 35-39 | 79.9 (75.8 - 83.9) | 73.4 (64.7 - 82.1) | 79.4 (70.4 - 88.5) | 73.9 (65.1 - 82.6) | 82.7 (73.5 - 92.0) | 90.0 (80.4 - 99.6) |
| 40-44 | 148.7 (143.5 - 153.8) | 128.2 (117.6 - 138.8) | 135.8 (124.8 - 146.8) | 156.7 (144.9 - 168.5) | 146.7 (135.3 - 158.1) | 176.0 (163.5 - 188.4) |
| 45-49 | 191.3 (185.4 - 197.2) | 172.1 (159.6 - 184.6) | 186.0 (173.0 - 199.0) | 183.7 (170.8 - 196.6) | 207.1 (193.4 - 220.8) | 207.6 (193.9 - 221.3) |
| 50-54 | 161.8 (156.3 - 167.2) | 142.9 (131.5 - 154.3) | 164.9 (152.7 - 177.2) | 152.1 (140.4 - 163.9) | 155.5 (143.5 - 167.4) | 193.4 (180.2 - 206.7) |
| 55-59 | 152.6 (147.0 - 158.2) | 149.7 (137.4 - 162.1) | 159.6 (146.8 - 172.4) | 145.2 (133.0 - 157.4) | 150.3 (137.9 - 162.7) | 158.1 (145.3 - 170.8) |
| 60-64 | 134.0 (127.7 - 140.3) | 138.4 (124.1 - 152.7) | 118.4 (105.2 - 131.6) | 126.1 (112.5 - 139.8) | 128.0 (114.3 - 141.8) | 159.2 (143.9 - 174.5) |
| 65-69 | 126.5 (119.7 - 133.3) | 119.9 (105.1 - 134.6) | 118.9 (104.2 - 133.6) | 116.1 (101.5 - 130.6) | 127.5 (112.2 - 142.7) | 150.2 (133.7 - 166.7) |
| 70-74 | 96.6 (90.4 - 102.7) | 105.1 (90.8 - 119.5) | 90.6 (77.3 - 104.0) | 93.7 (80.2 - 107.3) | 83.9 (71.0 - 96.7) | 109.5 (94.8 - 124.1) |
| 75-79 | 68.5 (62.8 - 74.3) | 83.5 (69.3 - 97.8) | 57.3 (45.4 - 69.1) | 58.4 (46.5 - 70.3) | 64.7 (52.2 - 77.3) | 78.7 (64.9 - 92.6) |
| 80-84 | 47.2 (41.2 - 53.3) | 53.2 (39.0 - 67.4) | 41.7 (28.9 - 54.5) | 35.0 (23.4 - 46.7) | 48.0 (34.5 - 61.6) | 58.1 (43.1 - 73.0) |
| 85+ | 30.9 (25.2 - 36.7) | 27.5 (15.7 - 39.2) | 24.4 (12.8 - 36.0) | 32.9 (19.8 - 46.1) | 32.9 (19.7 - 46.0) | 37.0 (23.0 - 50.9) |
| Mortality |  |  |  |  |  |  |
| 20-24 | 0.1 (0.0 - 0.2) | 0.3 (0.0 - 0.9) | 0.0 (0.0 - 0.0) | 0.0 (0.0 - 0.0) | 0.0 (0.0 - 0.0) | 0.0 (0.0 - 0.0) |
| 25-29 | 0.6 (0.2 - 1.0) | 1.0 (0.0 - 2.1) | 0.3 (0.0 - 1.0) | 0.0 (0.0 - 0.0) | 0.3 (0.0 - 1.0) | 1.3 (0.0 - 2.7) |
| 30-34 | 2.1 (1.5 - 2.8) | 3.2 (1.4 - 5.0) | 2.7 (1.0 - 4.3) | 1.3 (0.2 - 2.5) | 2.4 (0.8 - 4.0) | 1.1 (0.0 - 2.1) |
| 35-39 | 5.4 (4.3 - 6.4) | 6.7 (4.1 - 9.3) | 5.4 (3.0 - 7.7) | 6.7 (4.1 - 9.3) | 4.8 (2.6 - 7.1) | 3.2 (1.4 - 5.0) |
| 40-44 | 9.0 (7.8 - 10.3) | 11.4 (8.2 - 14.5) | 7.7 (5.1 - 10.3) | 9.4 (6.6 - 12.3) | 8.3 (5.6 - 11.0) | 8.3 (5.6 - 11.0) |
| 45-49 | 14.3 (12.7 - 15.9) | 18.3 (14.2 - 22.4) | 9.9 (6.9 - 12.9) | 13.0 (9.5 - 16.4) | 16.0 (12.2 - 19.8) | 14.1 (10.5 - 17.7) |
| 50-54 | 16.4 (14.6 - 18.1) | 23.0 (18.4 - 27.6) | 14.7 (11.1 - 18.4) | 15.7 (11.9 - 19.4) | 19.0 (14.8 - 23.1) | 9.5 (6.6 - 12.4) |
| 55-59 | 18.8 (16.8 - 20.7) | 26.3 (21.1 - 31.5) | 18.1 (13.8 - 22.4) | 15.2 (11.2 - 19.1) | 19.1 (14.7 - 23.6) | 15.2 (11.2 - 19.1) |
| 60-64 | 17.8 (15.5 - 20.1) | 22.3 (16.6 - 28.0) | 17.7 (12.6 - 22.8) | 17.3 (12.2 - 22.4) | 15.4 (10.6 - 20.1) | 16.1 (11.3 - 21.0) |
| 65-69 | 17.3 (14.8 - 19.9) | 21.3 (15.1 - 27.6) | 15.6 (10.3 - 21.0) | 12.3 (7.6 - 17.1) | 17.1 (11.5 - 22.6) | 20.4 (14.3 - 26.5) |
| 70-74 | 18.0 (15.3 - 20.7) | 25.0 (18.0 - 32.0) | 19.5 (13.3 - 25.6) | 14.3 (9.0 - 19.7) | 13.3 (8.2 - 18.4) | 17.9 (12.0 - 23.8) |
| 75-79 | 17.4 (14.5 - 20.3) | 20.9 (13.8 - 28.0) | 17.8 (11.2 - 24.4) | 17.8 (11.2 - 24.4) | 14.0 (8.1 - 19.8) | 16.5 (10.2 - 22.8) |
| 80-84 | 21.2 (17.2 - 25.3) | 27.6 (17.4 - 37.8) | 20.4 (11.4 - 29.3) | 17.0 (8.9 - 25.1) | 15.0 (7.4 - 22.6) | 26.0 (16.0 - 36.0) |
| 85+ | 26.8 (21.5 - 32.2) | 26.2 (14.7 - 37.6) | 24.4 (12.8 - 36.0) | 37.0 (23.1 - 51.0) | 13.7 (5.2 - 22.2) | 32.9 (19.7 - 46.0) |
