# Supplementary material for: Contrasting income-based inequalities in incidence and mortality of breast cancer in Korea, 2006-2015
Source: Epidemiol Health. 2024 Sep 11;46:e2024074. doi: 10.4178/epih.e2024074 (PMC11826041; doi:10.4178/epih.e2024074)
Supplement: Supplementary Material 7. — The age-standardized incidence, mortality rates, and mortality-to-incidence ratios of breast cancer by income quintile between 2006 and 2015 in Korea [file epih-46-e2024074-Supplementary-7.docx]

Supplementary Material 7. The age-standardized incidence, mortality rates, and mortality-to-incidence ratios of breast cancer by income quintile between 2006 and 2015 in Korea

|  | Total | Q1 (lowest) | Q2 | Q3 | Q4 | Q5 (highest) |
| --- | --- | --- | --- | --- | --- | --- |
| Incidence | | | | | | |
| 2006 | 70.1 (68.9 - 71.4) | 76.9 (74.0 - 79.9) | 60.5 (57.9 - 63.1) | 64.9 (62.2 - 67.6) | 69.8 (67.0 - 72.6) | 78.5 (75.5 - 81.5) |
| 2007 | 73.2 (72.0 - 74.5) | 68.5 (65.8 - 71.2) | 69.2 (66.4 - 71.9) | 67.4 (64.7 - 70.1) | 75.4 (72.6 - 78.3) | 85.5 (82.4 - 88.6) |
| 2008 | 76.3 (75.1 - 77.6) | 72.1 (69.3 - 74.9) | 68.8 (66.1 - 71.5) | 73.9 (71.1 - 76.7) | 77.1 (74.3 - 80.0) | 89.7 (86.6 - 92.8) |
| 2009 | 80.1 (78.8 - 81.4) | 76.3 (73.5 - 79.1) | 70.7 (68.0 - 73.4) | 76.2 (73.4 - 79.0) | 82.7 (79.8 - 85.6) | 94.7 (91.6 - 97.8) |
| 2010 | 83.1 (81.8 - 84.4) | 76.8 (74.0 - 79.7) | 80.1 (77.2 - 83.0) | 78.0 (75.1 - 80.8) | 83.8 (80.8 - 86.7) | 96.9 (93.7 - 100.0) |
| 2011 | 89.2 (87.9 - 90.6) | 82.9 (80.1 - 85.8) | 82.5 (79.6 - 85.3) | 87.3 (84.4 - 90.3) | 90.5 (87.5 - 93.5) | 103.0 (99.8 - 106.2) |
| 2012 | 89.4 (88.1 - 90.7) | 82.7 (79.8 - 85.5) | 80.6 (77.8 - 83.4) | 87.1 (84.2 - 90.0) | 90.7 (87.7 - 93.6) | 106.1 (102.9 - 109.4) |
| 2013 | 92.9 (91.5 - 94.2) | 88.1 (85.2 - 91.0) | 86.8 (83.9 - 89.7) | 91.1 (88.2 - 94.1) | 93.3 (90.3 - 96.4) | 104.9 (101.7 - 108.1) |
| 2014 | 96.7 (95.3 - 98.1) | 90.9 (88.0 - 93.9) | 92.4 (89.4 - 95.4) | 93.6 (90.6 - 96.6) | 96.7 (93.7 - 99.7) | 109.8 (106.6 - 113.1) |
| 2015 | 100.5 (99.2 - 101.9) | 93.8 (90.9 - 96.8) | 97.0 (93.9 - 100.0) | 96.9 (93.9 - 99.9) | 101.4 (98.3 - 104.5) | 113.7 (110.5 - 117.0) |
| Mortality | | | | | | |
| 2006 | 9.2 (8.8-9.7) | 13.4 (12.1-14.6) | 7.6 (6.7-8.6) | 7.7 (6.7-8.7) | 9.4 (8.3-10.4) | 8.0 (7.0-9.0) |
| 2007 | 9.4 (8.9-9.8) | 13.7 (12.4-14.9) | 7.7 (6.8-8.6) | 8.1 (7.2-9.1) | 8.4 (7.4-9.3) | 8.9 (7.9-9.9) |
| 2008 | 9.4 (8.9-9.8) | 14.1 (12.9-15.4) | 8.1 (7.1-9.0) | 8.4 (7.4-9.3) | 8.7 (7.7-9.6) | 7.6 (6.7-8.5) |
| 2009 | 10.0 (9.6-10.5) | 13.3 (12.1-14.5) | 9.2 (8.2-10.1) | 8.9 (7.9-9.9) | 9.8 (8.8-10.9) | 9.0 (8.0-9.9) |
| 2010 | 9.6 (9.2-10.1) | 14.6 (13.4-15.9) | 8.0 (7.1-8.9) | 9.0 (8.1-10.0) | 8.2 (7.3-9.1) | 8.3 (7.3-9.2) |
| 2011 | 10.1 (9.6-10.5) | 14.6 (13.4-15.8) | 8.1 (7.2-9.0) | 9.2 (8.3-10.2) | 9.5 (8.5-10.4) | 8.9 (7.9-9.8) |
| 2012 | 9.8 (9.4-10.3) | 14.3 (13.1-15.5) | 8.4 (7.5-9.3) | 8.2 (7.3-9.1) | 9.1 (8.2-10.1) | 9.0 (8.1-10.0) |
| 2013 | 10.6 (10.2-11.1) | 14.1 (12.9-15.2) | 9.4 (8.4-10.3) | 9.5 (8.6-10.5) | 10.4 (9.4-11.4) | 9.7 (8.7-10.7) |
| 2014 | 10.4 (10.0-10.9) | 13.7 (12.5-14.8) | 8.7 (7.8-9.6) | 9.5 (8.6-10.4) | 10.8 (9.8-11.9) | 9.5 (8.5-10.4) |
| 2015 | 10.6 (10.1-11.0) | 13.8 (12.7-14.9) | 9.7 (8.8-10.6) | 9.7 (8.8-10.7) | 10.1 (9.1-11.1) | 9.4 (8.5-10.4) |
| Mortality-to-incidence ratio | | |  |  |  |  |
| 2006 | 0.13 (0.12 - 0.14) | 0.17 (0.16 - 0.19) | 0.13 (0.11 - 0.14) | 0.12 (0.10 - 0.13) | 0.13 (0.12 - 0.15) | 0.10 (0.09 - 0.12) |
| 2007 | 0.13 (0.12 - 0.13) | 0.20 (0.18 - 0.22) | 0.11 (0.10 - 0.13) | 0.12 (0.11 - 0.14) | 0.11 (0.10 - 0.12) | 0.10 (0.09 - 0.12) |
| 2008 | 0.12 (0.12 - 0.13) | 0.20 (0.18 - 0.21) | 0.12 (0.10 - 0.13) | 0.11 (0.10 - 0.13) | 0.11 (0.10 - 0.13) | 0.08 (0.07 - 0.09) |
| 2009 | 0.13 (0.12 - 0.13) | 0.17 (0.16 - 0.19) | 0.13 (0.11 - 0.14) | 0.12 (0.10 - 0.13) | 0.12 (0.11 - 0.13) | 0.09 (0.08 - 0.11) |
| 2010 | 0.12 (0.11 - 0.12) | 0.19 (0.17 - 0.21) | 0.10 (0.09 - 0.11) | 0.12 (0.10 - 0.13) | 0.10 (0.09 - 0.11) | 0.09 (0.08 - 0.10) |
| 2011 | 0.11 (0.11 - 0.12) | 0.18 (0.16 - 0.19) | 0.10 (0.09 - 0.11) | 0.11 (0.09 - 0.12) | 0.10 (0.09 - 0.12) | 0.09 (0.08 - 0.10) |
| 2012 | 0.11 (0.10 - 0.11) | 0.17 (0.16 - 0.19) | 0.10 (0.09 - 0.12) | 0.09 (0.08 - 0.11) | 0.10 (0.09 - 0.11) | 0.09 (0.08 - 0.09) |
| 2013 | 0.11 (0.11 - 0.12) | 0.16 (0.15 - 0.17) | 0.11 (0.10 - 0.12) | 0.10 (0.09 - 0.12) | 0.11 (0.10 - 0.12) | 0.09 (0.08 - 0.10) |
| 2014 | 0.11 (0.10 - 0.11) | 0.15 (0.14 - 0.16) | 0.09 (0.08 - 0.10) | 0.10 (0.09 - 0.11) | 0.11 (0.10 - 0.12) | 0.09 (0.08 - 0.10) |
| 2015 | 0.11 (0.10 - 0.11) | 0.15 (0.13 - 0.16) | 0.10 (0.09 - 0.11) | 0.10 (0.09 - 0.11) | 0.10 (0.09 - 0.11) | 0.08 (0.07 - 0.09) |
